# W-Band Beam-Tilted H-Plane Horn Array Antenna with Wideband Integrated Waveguide Feed Network Based on MMPTE

**DOI:** 10.3390/mi14020259

**Published:** 2023-01-19

**Authors:** Yun Zhao, Fan Ye, Sheng Li, Ai-Zhong Wang, Jiang-Qiao Ding

**Affiliations:** 1 Jiangsu Collaborative Innovation Center of Atmospheric Environment and Equipment Technology (CICAEET), School of Electronic & Information Engineering, Nanjing University of Information Science and Technology, Nanjing 210044, China; 2 National Key Laboratory of Science and Technology on Space Microwave, China Academy of Space Technology, Xi’an 710100, China

**Keywords:** H-plane horn antenna, beam steering, broadband feed network, MMPTE, terahertz

## Abstract

A W-band H-plane horn array antenna with tilted radiation beam based on waveguide structure is proposed in this paper. The designed antenna array consists of four H-plane antenna elements and a broadband feed network. The distribution of excitations is determined by the theory of maximum power transmission efficiency (MMPTE). A multiple branch coupler, two T-junctions and three fixed phase shifters are employed to construct the feed network, which can generate the required amplitude and phase in broadband frequency range from 80 GHz to 100 GHz. The computer numerical control (CNC) milling machines technology is employed to machine the feed network and antenna. All measured and simulated results are in good agreement, which verify the feasibility of the theory of MMPTE to generate a radiation beam directed to any angle from −35° to 35° with suitable excitation provided by the proposed feed network in this paper.

## 1. Introduction

With the rapid development of wireless communication technology, low frequency spectral resources are becoming more and more exhausted; the millimeter and terahertz wave have thus attracted great attention due to their wide bandwidth, high date rate, large user capacity and high imaging resolution, etc. Nevertheless, the millimeter and terahertz technologies have to face some challenges, the important one of which is multipath effect or multipath interference between adjacent wireless channels, which will reduce the quality of signal [1]. The tilted-beam antenna can be employed to cope with the multipath effect and improve the communication quality to some extent [2].

Various methods have been proposed to realize beam-tilted antenna in the millimeter and terahertz wave band. The most conventional method is mechanical beam tilting [3]. However, the rotation parts would increase system complexity and reduce system stability. Another method is to load metamaterial, such as in [4], where high refractive-index metamaterial unit cells were loaded along the radiation direction of the planar dipole antenna. The antenna realized beam tilting of 30° with the peak gain of 12 dBi over 57–64 GHz. In [5], the multilayer frequency selective surface (FSS) structure was placed below the Vivaldi antenna to realize a beam tilting angle of 38° with peak gain of 9 dBi and SLL of −8 dB at 28 GHz. Leaky wave antenna is also used to realize titled-beam; the most common existing type is waveguide slot antenna [6]. What is more, some electronic element also can be adopted to tilt the antenna beam, such as pin diodes, varactor diodes and MEMS switches [7,8,9,10]. However, these electronic elements have large insertion loss and are not even available at high frequencies, such as THz wave band. Some passive circuits can also be used to realize tilted-beam antenna [11,12]. In [11], a butler network was designed to drive horn antenna and it obtained the maximum beam tilting angle with 42° and the peak gain with 11.2 dBi at 30 GHz. The traditional method to calculate the antenna pointing angle depends on the array factor. The principle of this method is to calculate the excitation distribution of array elements through the preset pattern and array element spacing. The excitation distribution of array will be obtained with difficultly or inaccurately when the array elements or element spacing are different. Another method is pattern synthesis, which employs some algorithms such as Woodward-Lawson algorithm, FFT or the Particle Swarm Optimization algorithm to solve the excitation of antenna array. Nevertheless, this method runs into a stone wall when the complex antenna structure cannot be expressed mathematically. What is more, the complex iterative process is also inefficient and very time-consuming for massive-element antenna arrays. The Method of Maximum Power Transmission Efficiency (MMPTE) can be used to solve these problems [13]. The MMPTE is characterized by reducing a field synthesis problem into a circuit problem and it can be used to realize the beam control of any complex antenna structure. Besides, it sets the PTE (Power Transmission Efficiency) between the transmitting and receiving antenna as the optimization goal, so the biggest gain of array can be achieved. The MMPTE has been used to realize the beam control of microstrip antenna array in microwave frequency band in [14,15]. In [14], a power transmission system was established, which was formed by an unequally spaced four-element transmitting patch array and three receiving dipoles, a flat-top radiation pattern in far-field region with 45° covering range was achieved at 5.4 GHz. In [15], a six-element printed dipole array was arranged as transmitting antenna, and the receiving antenna was placed at one end of it; the array with end-fire characteristic was achieved at 2.45 GHz. Nevertheless, its applications are all limited to the microwave band and microstrip antenna. In this paper, this method will be applied in millimeter and terahertz wave for the first time.

As frequency increases, the thickness of substrate becomes bigger relative to the wavelength; most of the energy is limited in the dielectric substrate and cannot be radiated out. Therefore, the power loss for microstrip antenna cannot be ignored in THz band. The waveguide structure is criticized for its large size and bulkiness in low frequency. However, these shortcomings are no longer prominent in high frequency band. In addition, the waveguide structure can easily be processed by current CNC milling machines technology. For this reason, the hollow waveguide structure becomes more practical to build antenna at high frequency for its advantages of low loss, high power capacity, easy fabrication, stable structure, etc. The horn antenna is selected as an array element for it has a wide bandwidth and high radiation efficiency. At the same time, to avoid the influence of large element spacing on radiation pattern, the H-plane horn antenna is selected as an element for it has minimum element spacing when it arranges along narrow edges. What is more, the H-plane horn antenna has wide E-plane beam, which means the H-plane horn array antenna can realize relatively larger beam tilted angle.

In this paper, four H-plane horn antennas are used to form a beam-tilted array in W-band. A feed network is proposed to obtain required excitation to realize the tilted beam based on the theory of MMPTE. To generate stable distributions of the excitation amplitude and phase in broadband, the proposed feed network consists of a directional coupler, two T-junctions and three phase shifters. All structures are based on standard waveguide WR-10. The proposed antenna array can achieve the radiation beam tilt-angle within 31–36° in the wide working frequency band from 80 GHz to 100 GHz. The proposed antenna is manufactured by the computer numerical control (CNC) milling machine for its high precision machining and low price. The feed network and antenna array are verified by simulation and measurement.

## 2. Design Method of Array Antenna

**Figure 1:**
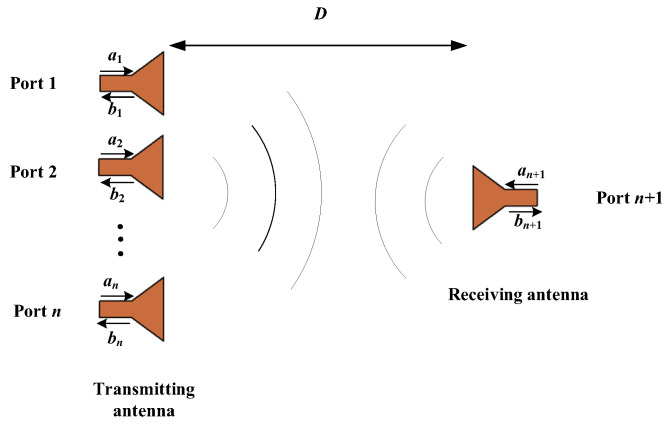
The basic model of power transmission system.

Consider a system consisting of an array of N elements and a testing (receiving) antenna. The distance of between transmitting array and receiving antenna is D, as shown in Figure 1. The whole system can be regard as a N + 1 ports network, and can be described by the scattering matrix as follows:

(1) $$\left[\begin{array}{c} \left[b_{t}\right]\\ \left[b_{r}\right] \end{array}\right]=\left[\begin{array}{cc} \left[S_{tt}\right] & \left[S_{tr}\right]\\ \left[S_{rr}\right] & \left[S_{rt}\right] \end{array}\right]\left[\begin{array}{c} \left[a_{t}\right]\\ \left[a_{r}\right] \end{array}\right]$$

where

(2) $$\left[a_{t}\right]={\left[a_{1},a_{2},\cdots a_{n}\right]}^{T}$$

(3) $$\left[a_{r}\right]={\left[a_{n+1},\right]}^{T}$$

(4) $$\left[b_{t}\right]={\left[b_{1},b_{2},\cdots b_{n}\right]}^{T}$$

(5) $$\left[b_{r}\right]={\left[b_{n+1},\right]}^{T}$$

are the normalized incident and reflected waves. The subscript *t* is used to designate the transmitting antenna array, and the subscript *r* for the receiving (testing) antenna. The power transmission efficiency *T_array_* between the transmitting antenna array and the receiving antenna is defined as the ratio of the power delivered to the load of the receiving antenna to the input power to the antenna array.

(6) $$T_{array}=\frac{\frac{1}{2}\left({\left|\left[b_{r}\right]\right|}^{2}-{\left|\left[a_{r}\right]\right|}^{2}\right)}{\frac{1}{2}\left({\left|\left[a_{t}\right]\right|}^{2}-{\left|\left[b_{t}\right]\right|}^{2}\right)}\;\;$$

If the whole system is matched, the optimal excitation distribution [$a_{t}$] of the antenna array, which maximizes the power transmission efficiency, satisfies:

(7) $$\left[A\right]\left[a_{t}\right]=T\left[a_{t}\right]\;$$

where $\left[A\right]={\left[{\bar{S}}_{rt}\right]}^{T}S_{rt}$. The Equation (7) is an algebraic eigenvalue equation, and it has only one positive eigenvalue. The positive eigenvalue is the maximum power transmission efficiency between the transmitting array and receiving array. The corresponding eigenvector is the optimized distribution of excitations (ODE) for the transmitting array. Each element in the eigenvector is a complex number. The modulus of each element of eigenvector correspond to the amplitude distributions of array, and the vector angle correspond to the phase distributions.

**Figure 2:**
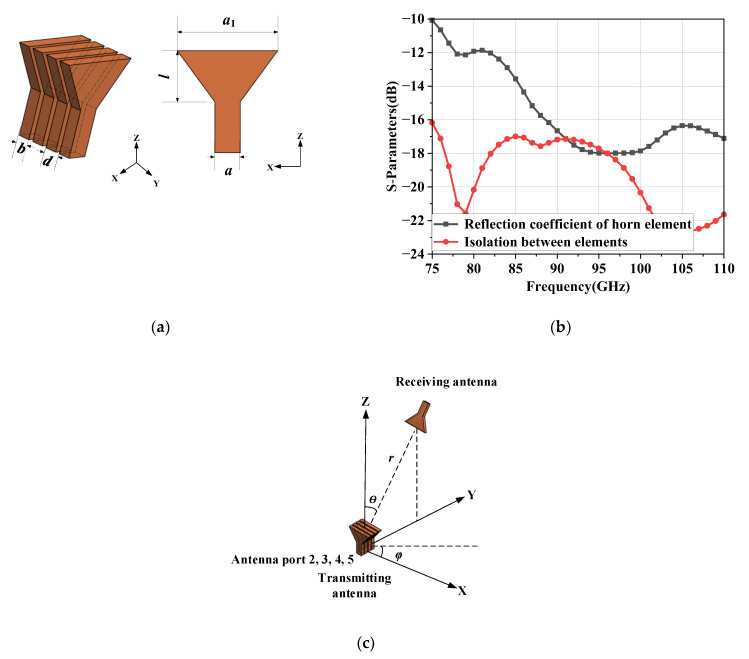
The diagram of (**a**) proposed H-plane horn array antenna (*b* = 1.27 mm, *d* = 1.77 mm, *a*_1_ = 10 mm, *l* = 5 mm, *a* = 2.54 mm); (**b**) the simulated reflection coefficient of horn element and simulated isolation between elements; (**c**) the power transmission system.

The H-plane horn antenna is selected as the array element for its simple structure, low profile in H-plane and wide E-plane radiation beam width. The dimensions of the element and the array configuration are shown in Figure 2a. The feed waveguide is standard waveguide WR-10 (2.54 mm × 1.27 mm). The element spacing is set to 1.77 mm (about 0.55 λ, λ is the space wavelength of 94 GHz) and the wall thickness of 0.5 mm between the elements is retained for avoiding the deformation of metal walls during CNC machining. The simulated reflection coefficient and isolation for horn elements are shown in Figure 2b, the reflection coefficient below −10 dB and the isolation below −15 dB in the frequency band from 75 GHz to 110 GHz.

Based on the theory of MMPTE introduced above, a power transmission system is established in Figure 2c. The power transmission system consists of four transmitting antennas and one receiving antenna. The parameter *r* represents the distance between the transmitting antenna and receiving antenna. The parameter *θ* and *φ* represent the azimuth angle between the transmitting array antenna and receiving antenna. Placing the receiving antenna at (*r*, *θ*, *φ*) = (300 mm, 20°, 90°), (300 mm, 35°, 90°) and (300 mm, 45°, 90°) respectively (the *r* = 300 mm is to ensure far field), the whole system is simulated with ANSYS HFSS 2020 R1, which generates the scattering parameters. The scattering parameters can be substituted into Equation (7). The optimized distribution of excitations (ODE) $\left[a_{t}\right]$ is listed in Table 1.

**Figure 3:**
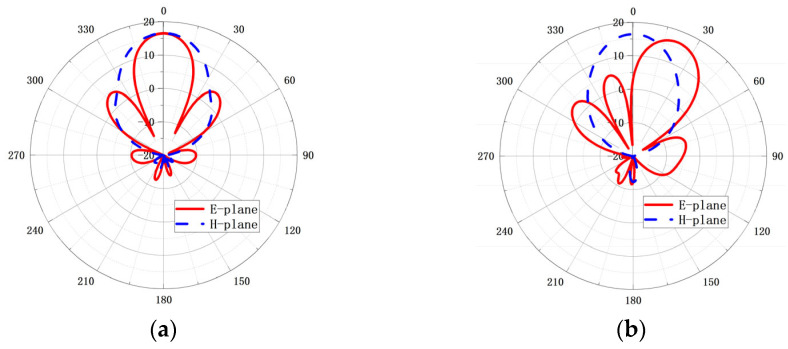

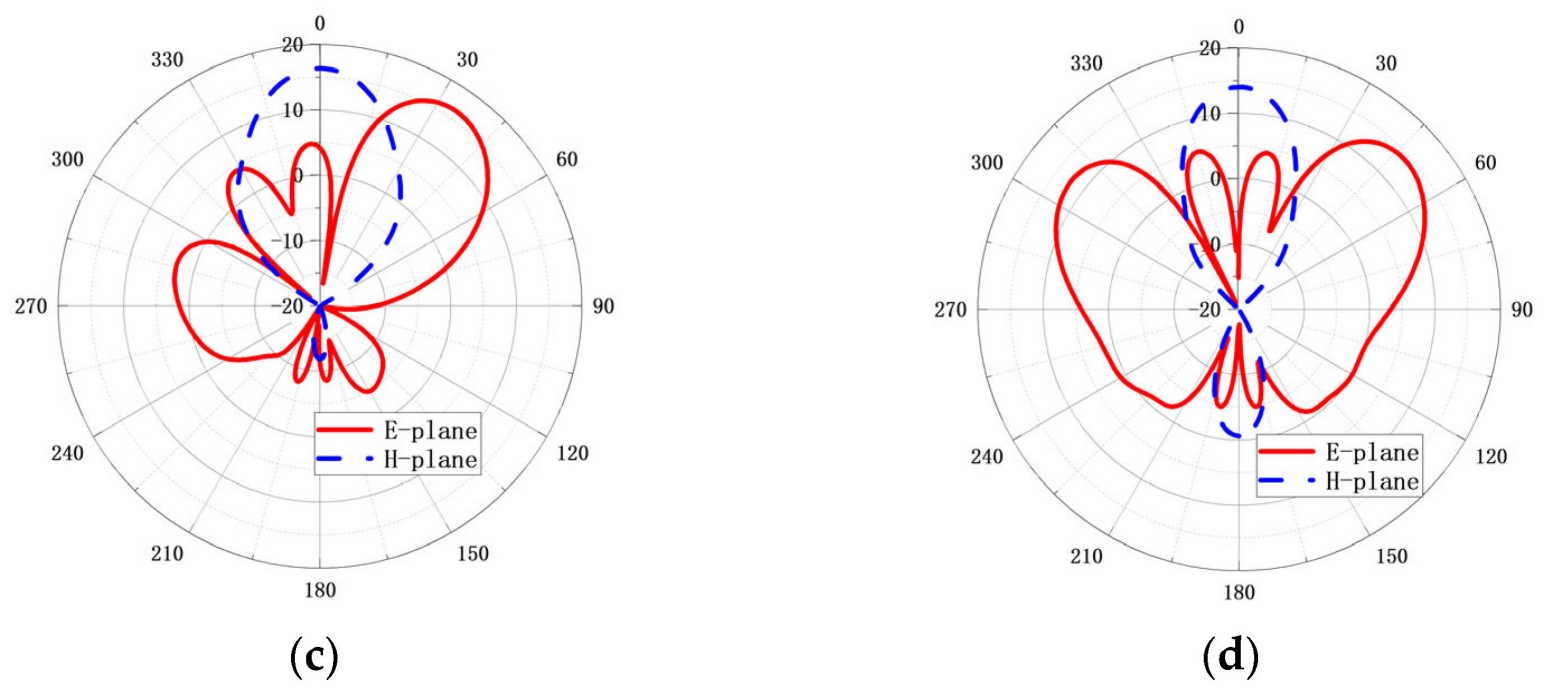
The simulated radiation patterns of the proposed H-plane horn array antenna at 94 GHz, (**a**) the radiation pattern for 0° tilt-angle; (**b**) the radiation pattern for 20° tilt-angle; (**c**) the radiation pattern for 35° tilt-angle; (**d**) the radiation pattern for 45° tilt-angle.

**Figure 4:**
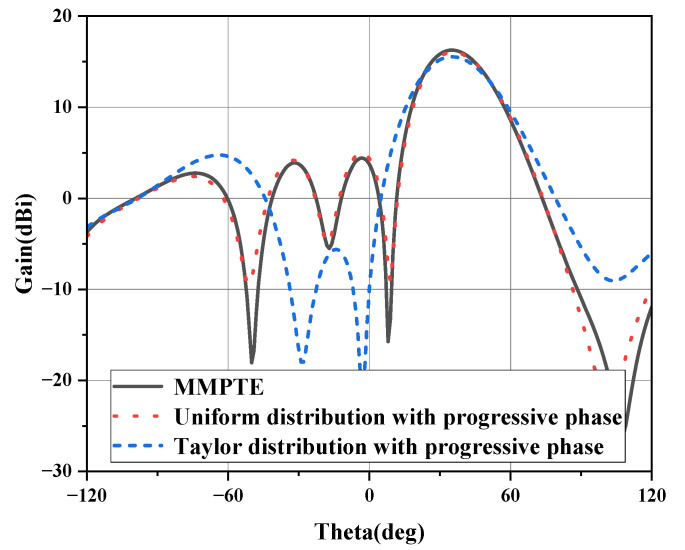
The comparison of radiation pattern with different excitation distributions at 94 GHz.

The simulated radiation patterns for different beam tilt-angles are shown in Figure 3. The ODE for the 0° tilt-angle has a uniform distribution of amplitude and phase, and the simulated gain of the array is 16.5 dB; the side lobe level is −12.2 dB; the E-plane beam-width is 33°. When the beam tilt-angle is 20°, the gain of array is still 16.5 dB; the side lobe level is −11.5 dB; the E-plane beam-width is 25°. When the beam tilt-angle is 35°, the gain is 16.3 dB; the side lobe level is −7.7 dB; the E-plane beam-width is 27°. However, when the main beam is directed to 45°, the radiation pattern deteriorated, the gain drops to 14.8 dB and the grating lobe appears in −55°. The comparison of the radiation patterns at 94 GHz for 35° tilt-angle is given in Figure 4 when the array is designed based on the MMPTE, uniform distribution with progressive phase and Taylor distribution with progressive phase, respectively. The targeted side lobes level for the Taylor distribution is −30 dB, and the calculated power distribution is 0.1, 0.4, 0.4, 0.1, respectively. To realize the 35° pointing angle, the progressive phase is 120°. It can be seen from the figure that the antenna fed by Taylor distribution has the lowest sidelobe level, but it also has the lowest gain of 15.5 dBi. The antennas based on MMPTE and uniform distribution have higher gain while the sidelobe level is larger. The gain of the antenna with uniform distribution is about 16 dBi and with the excitation distribution based on MMPTE is about 16.3 dBi. The MMPTE set the PTE (Power Transmission Efficiency) between the transmitting and receiving antenna as optimization goal, so it is easier to achieve higher gain. All of the radiation patterns shown in Figure 3 can be realized with corresponding feed network proposed below. The feed network for 35° tilt-angle will be designed as a proof and the design details will be presented in next section.

## 3. Design of Feed Network

**Figure 5:**
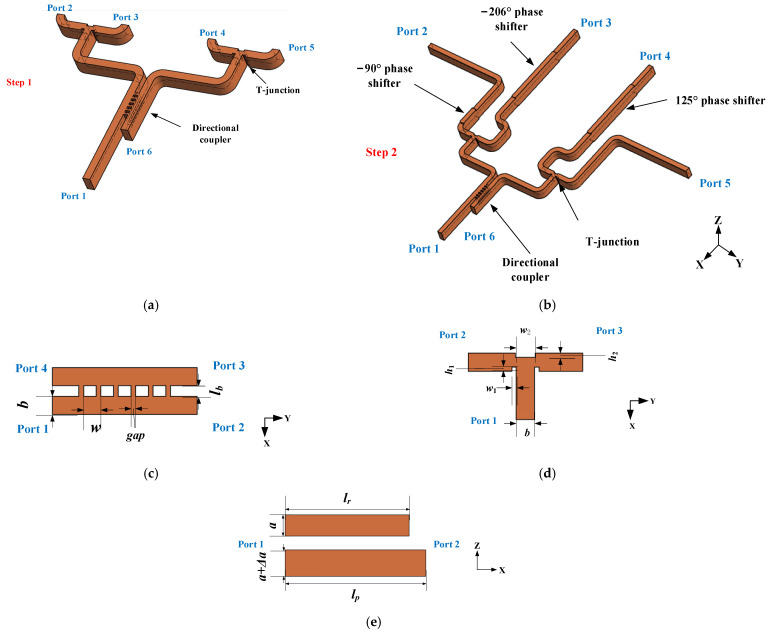
The structure of the proposed feed network, (**a**) the first step of feed network; (**b**) the second step of feed network; (**c**) the diagram and relevant dimensions of multiple directional coupler; (**d**) the diagram and relevant dimensions of T-junction; (**e**) the diagram and relevant dimensions of phase shifter, (*a* = 2.54 mm, *b =* 1.27 mm, *l_b_* = 0.77 mm, *w* = 0.78 mm, *gap* = 0.43 mm, *w*_1_ = 0.47 mm, *w*_2_ = 1.1 mm, *h*_1_ = 0.3 mm, *h*_2_ = 0.3 mm).

Based on the ODE shown in Table 1, a feed network will be designed to realize the radiation pattern beamed to 35° to validate the optimization method. The output amplitude and phase of the feed network are 0.3 ∠ 1°, 0.3 ∠ 125°, 0.2 ∠ −116°, 0.2 ∠ 0° approximately. Since the antennas are arranged along the E-plane, the feed network is also designed based on the E-plane structure. The diagram of proposed feed network is shown in Figure 5. The designed feed network consists of power divider and phase shifter. The power divider contains three passive components, a multiple branch directional coupler and two T-junctions. The phase shift is constituted by −90°, −206° and 125° broad fixed phase shifters. The whole network has six ports, among which port 1 is the input port, port 2 to port 5 are outputs, and port 6 is the isolation port of coupler. The design of feed network is divided into two steps. The first step is to realize power distribution by employ a directional coupler and two T-junctions. The second step is to compensate the output phase by three phase shifters. The diagrams for step 1 and step 2 are shown in Figure 5a and b. The layout of the output ports in step 2 for reserving space to assemble flange and measurement subsequently.

**Figure 6:**
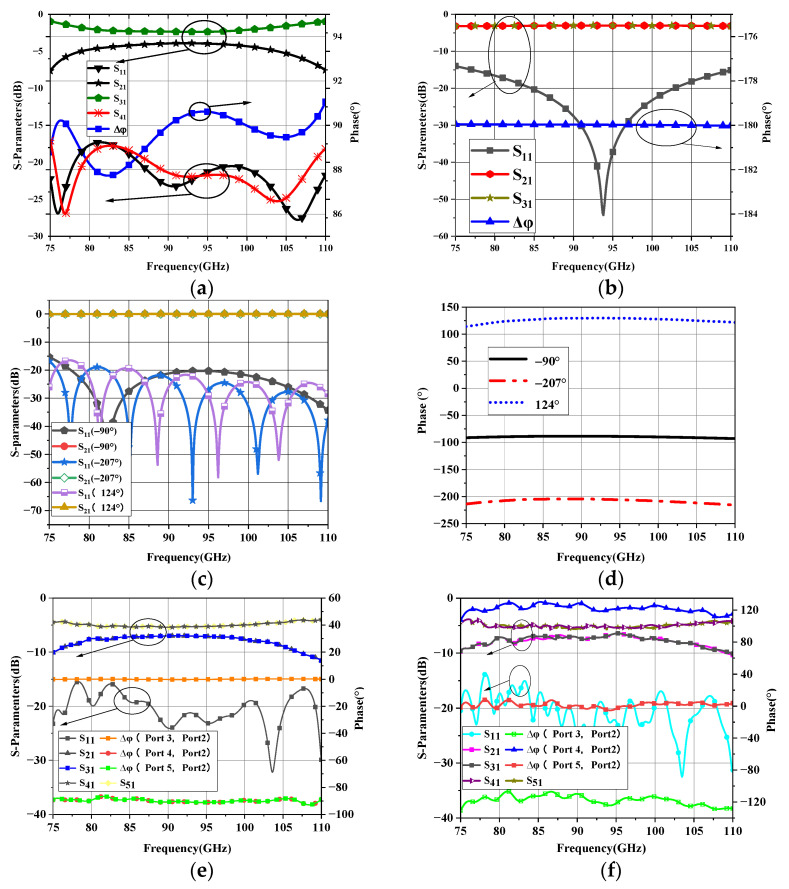
The simulated results of the proposed feed network, (**a**) the simulated s-parameters and phase imbalance of the proposed coupler; (**b**) the simulated s-parameters of the proposed T-junction; (**c**) the simulated s-parameters of the proposed phase shifter; (**d**) the simulated phase deviation of the proposed phase shifter; (**e**) the simulated s-parameters and output phase of the structure in step 1; (**f**) the simulated s-parameters and output phase of the structure in step 2.

The traditional multiple branch waveguide directional coupler are discussed in [16,17]. Generally, the branch has different heights (gap), which makes the manufacture difficult at high frequency. According to [18], unified branch height can be adopted to solve the problem of the low fabrication precision at high frequency. Therefore, a directional coupler with unified branch is employed in this paper. The directional coupler features a low amplitude imbalance $\left(\Delta A\right)$ and nearly 90° phase difference between the coupled and direct ports. It is worthy mention that any other coupling ratio can be achieved. As demonstrated in [18], when the number of branches increases, ∆*A* becomes better. However, with the increase of the number, the height of branch will become smaller meanwhile, which brings out unfeasibility of the machining of the coupler especially in high frequency band. Considering both the performance and machining, a unified 6-branch coupler is adopted in this paper. The diagram and relevant dimensions of the directional coupler with unified branch are shown in Figure 5c. The dimensions are optimized and the simulated results are shown in Figure 6a. The 2.2 dB coupler exhibits great isolation and S_11_ better than −15 dB, amplitude imbalance less than 0.3 dB, maximum phase imbalance less than 2.3°, and 21% bandwidth from 80 GHz to 100 GHz.

The E-plane T-junction is employed to realize 3 dB power divider in the broadband. To achieve good input impedance matching in a wider bandwidth, the rectangular groove is adopted in the symmetry plane of T-junction, the diagram of T-junction is shown in Figure 5d. Unlike the triangular wedge or ladder structure used in [19,20], the rectangular groove structure has less manufacturing difficultly and more feasibility of CNC-milling. The simulated results of the proposed T-junction are shown in Figure 6b. It can be seen that, the S_11_ is below −14 dB, the S_21_ and S_31_ are fairly close to −3 dB, and the phase difference between port 2 and port 3 is 180° with the phase fluctuation within 1 deg in the frequency band from 75 GHz to 110 GHz.

The widened rectangular waveguide fixed phase shifter was proposed in [21], which has the advantages of simple structure, wide bandwidth, low insertion loss, high power capacity, and large phase shift range. What is more, the structure can be processed by CNC-machining. Therefore, the structure is employed to realize the required phase difference in broadband. The proposed structure is shown in Figure 5e, where the length of the widen waveguide is *l_p_*, the waveguide width is a+Δa and the length and width of the reference standard WR-10 is *l_r_* and *a*, respectively. Assuming the electromagnetic wave enters these two waveguides with the same initial phase and the output phase are *φ*_1_ and *φ*_2_ respectively. The phase difference between these two output ports is Δφ.

(8) $$\Delta \varphi =\varphi_{2}-\varphi_{1}=\frac{2\pi \cdot l_{r}}{\lambda_{g}\left(a\right)}-\frac{2\pi \cdot l_{p}}{\lambda_{g}\left(a+\Delta a\right)}$$

where $\lambda_{g}\left(a\right)=\lambda /{\left(1-{\left(\lambda /2a\right)}^{2}\right)}^{1/2}$, *λ* is the space wavelength. The parameters *l_p_*, *l_r_* and Δ*a* are optimized by the electromagnetic simulation software, the optimized values of Δ*a* are 0.62 mm (−90°), 0.34 mm (−206°), 0.4 mm (125°), the *l_r_* are 0.49 mm (−90°), 14.8 mm (−206°), 15.7 mm (125°) and the *l_p_* are 5 mm (−90°), 16 mm (−206°), 17 mm (−125°). The results of simulated S-parameter and required phase difference are shown in Figure 6c,d. It can be seen that, the S_11_ is below −19 dB, the S_21_ is nearly close to 0 dB and the phase deviation is within ±5° in the frequency band from 78 GHz to 110 GHz of the proposed three phase shifters. The performances of the structure in step 1 are shown in Figure 6e, the phase difference between port 3 and 2, port 4 and 2, port 5 and 2 is 0°, −90°, −90°, respectively, and its fluctuation less than 2° from 80 GHz to 100 GHz. The output amplitude fluctuation less than 0.3 dB in the same frequency band. The performance of the structure in step 2 is shown in Figure 6f, the phase difference between port 3 and 2, port 4 and 2, port 5 and 2 is −116°, 125°, 1°, respectively, and its fluctuation less than 10° from 80 GHz to 100 GHz. The output amplitude fluctuation is less than 0.6 dB in the same frequency band.

**Figure 7:**
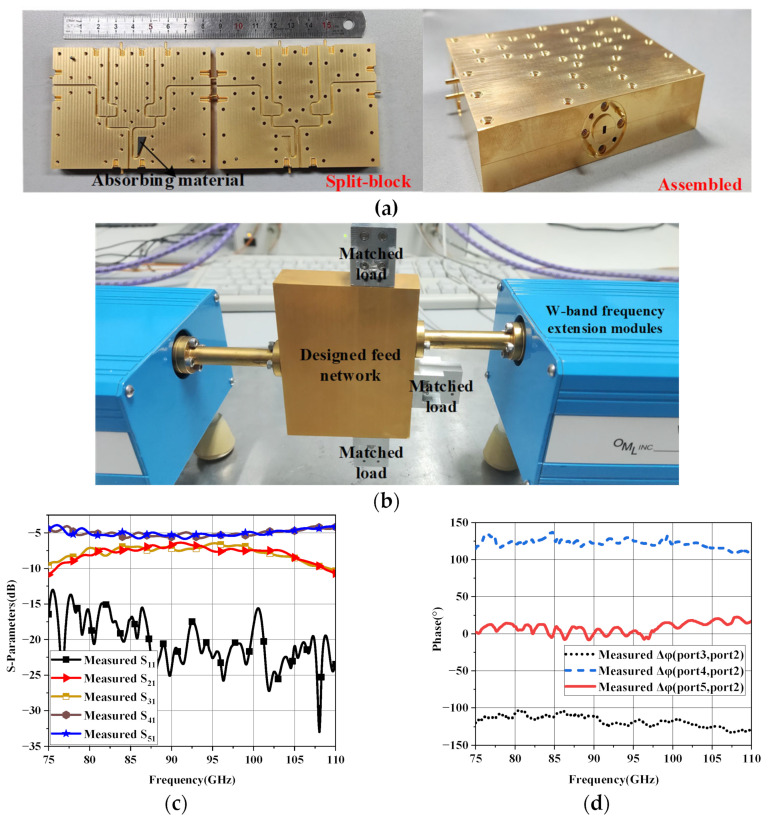
The results of the proposed feed network, (**a**) the photograph of feed network fabricated by E-plane split block CNC-machining; (**b**) the measured environment of proposed feed network; (**c**) the s-parameters results of proposed feed network; (**d**) the phase performance of proposed feed network.

The proposed feed network is manufactured by the computer numerical control (CNC) milling machines. In order to adapt to CNC machining, the minimum rounded corners radius is 0.25 mm. The manufactured gold-plated aluminum E-plane split blocks are shown in Figure 7a, the two layers are completely symmetrical. The thickness of each layer is 10 mm and the port 1 to port 5 in step 2 are configured with standard UG-387 flange to connect with Vector Network Analyzer (VNA), the absorbing material is introduced in the isolation port of the coupler (port 6 in Figure 5a of step 2) to absorb the reflected wave. Two layers of blocks are aligned by pins and fixed by screws. The fabricated feed network is measured by Agilent N5260A Vector Network Analyzer (VNA) with OML W-band frequency expansion module. The measurement environment is shown in Figure 7b, the measured ports are connected with VNA and the other ports are connected with W-band matched load. The measured results of the proposed feed network are shown in Figure 7c,d. The transmission coefficient between port 2 and 3 is −7 dB while it of port 4 and 5 is −5.2 dB, both of the fluctuations are less than 1 dB. The S_11_ is below −15 dB in the working frequency band from 80 GHz to 100 GHz. The phase differences between port 3 and port 2, port 4 and port 2, port 5 and port 2 are −116°, 125° and 1°, respectively. In addition, the measured phase fluctuation is within ±15° in the frequency band from over the same working frequency band.

## 4. Discussion of the Proposed Antenna Array

**Figure 8:**
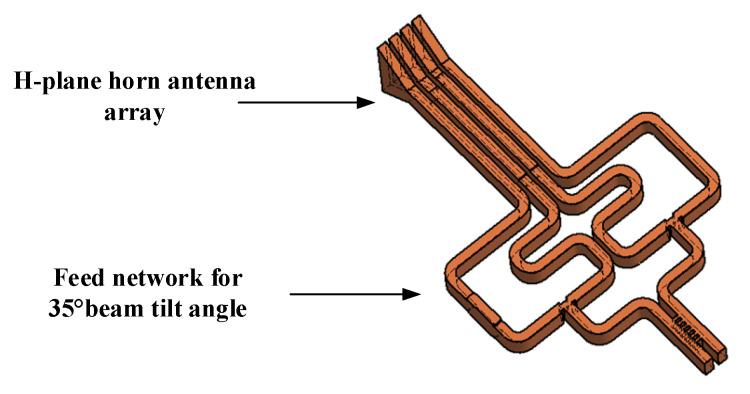
The structure of the whole array antenna integrated with feed network.

**Figure 9:**
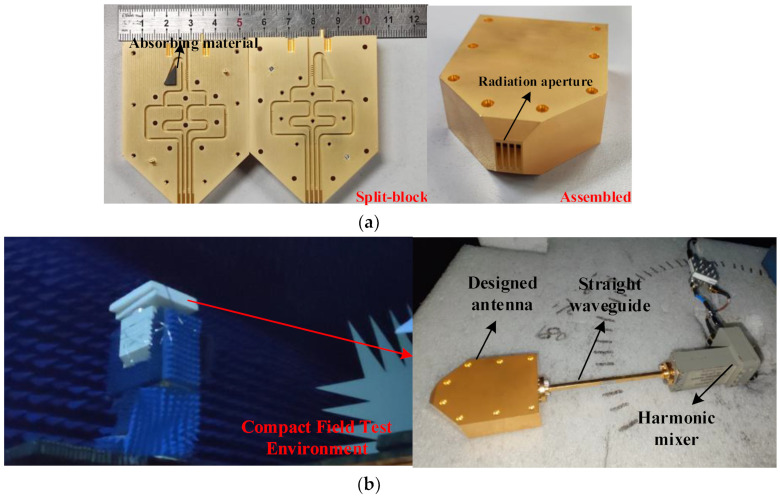
The photograph of (**a**) antenna fabricated by E-plane split block CNC-machining; (**b**) compact field measure environment.

According to the measured results shown in the Section 3. The designed feed network can output stable amplitude and phase in the broadband. The layout of feed network in Section 3 is for assembling the flanges. To connect with the horn array which has 1.77 mm element spacing, the layout of feed network is modified without changing the dimensions of component. The integration of array and feed network to realize the 35° tilt angle is shown in Figure 8. The antenna is manufactured by CNC-milling, and the manufactured gold-plated aluminum E-plane split blocks and the ultimate block are shown in Figure 9a. The UG-387 flange is configured in the input port of antenna and the absorbing material is placed in the isolated port. The blocks are aligned with pins and then fixed with screws. It is taken notice that the radiation aperture is sliced off partly to reduce the effects of surface wave on radiation. The radiation performance of the designed antenna is measured in compact field test range, and the test environment is shown in Figure 9b. The proposed antenna is connected to the mixer through a WR-10 straight waveguide, then the mixer is connected to RF receiver through coaxial cable, and the RF signal is produced by W-band active multiplier. The radiation characteristic of the proposed antenna is measured with a W-band standard gain horn antenna.

**Figure 10:**
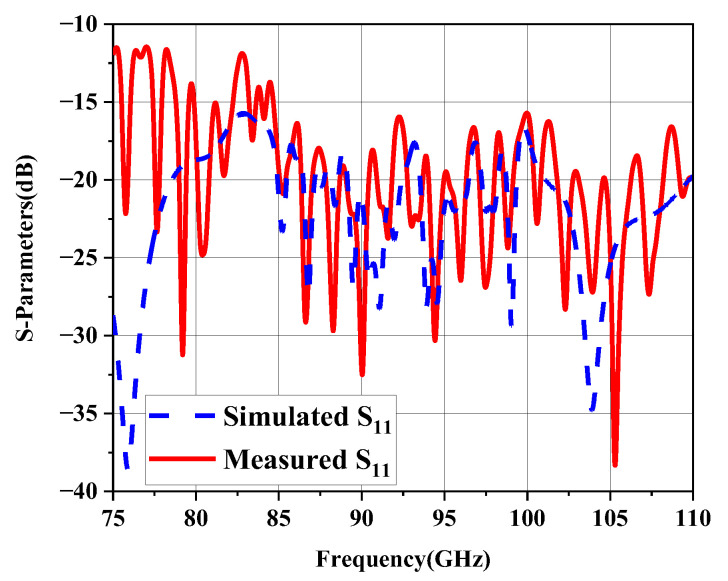
The simulated and measured reflection coefficient of the proposed array antenna.

The reflection coefficient of the proposed antenna is measured by VNA and the results are shown in Figure 10. The simulated S_11_ is below −15 dB, and the measured one is below −12 dB from 75 GHz to 110 GHz, the simulated and measured results consistent generally.

**Figure 11:**
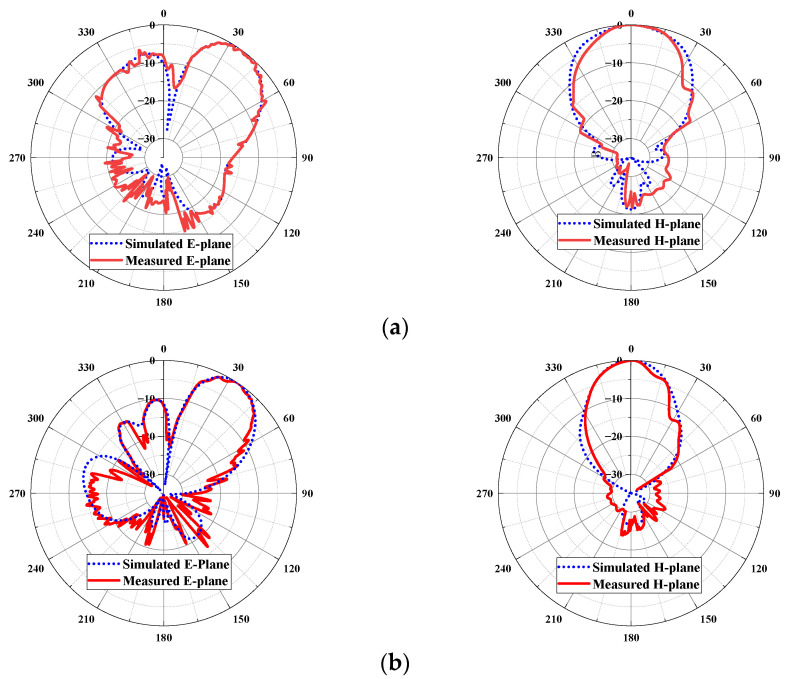

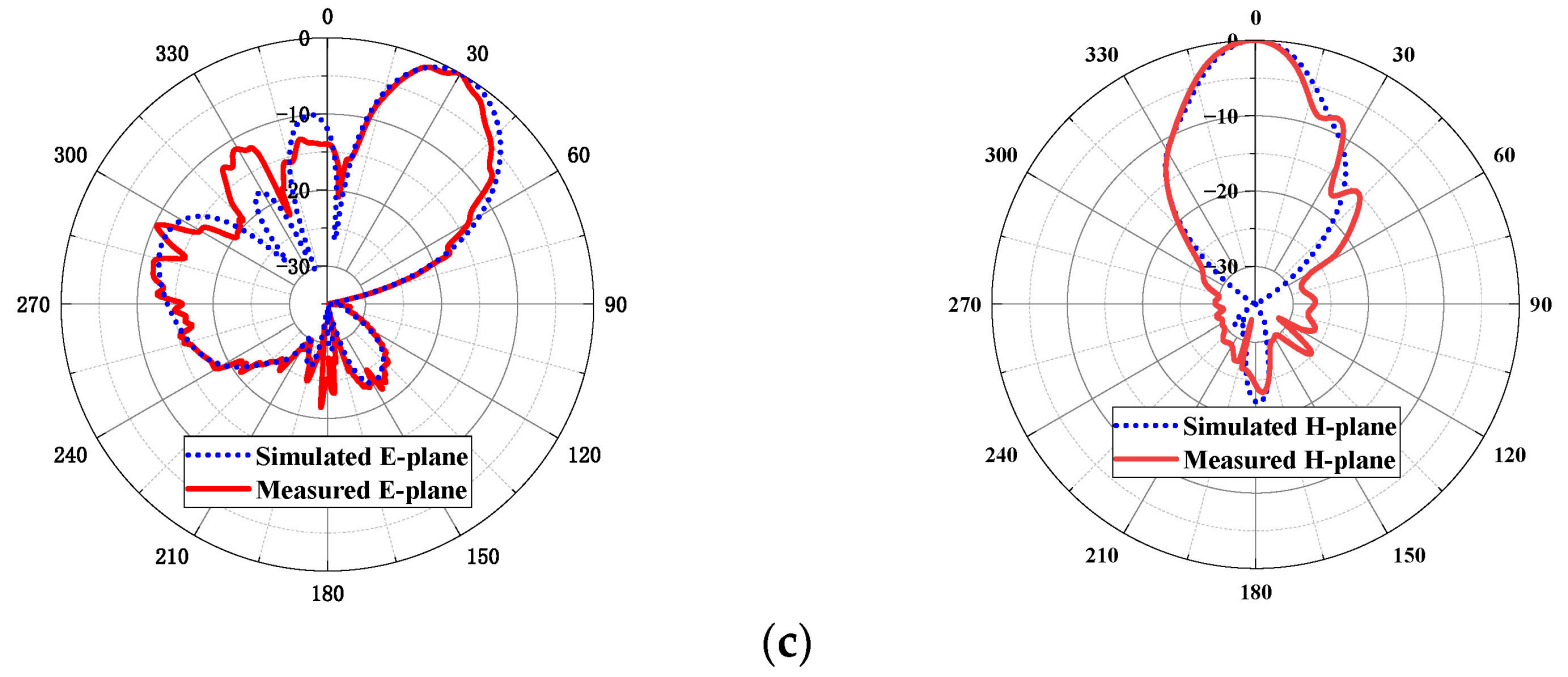
The simulated and measured radiation patterns of the proposed antenna, (**a**) 80 GHz; (**b**) 94 GHz; (**c**) 100 GHz.

**Figure 12:**
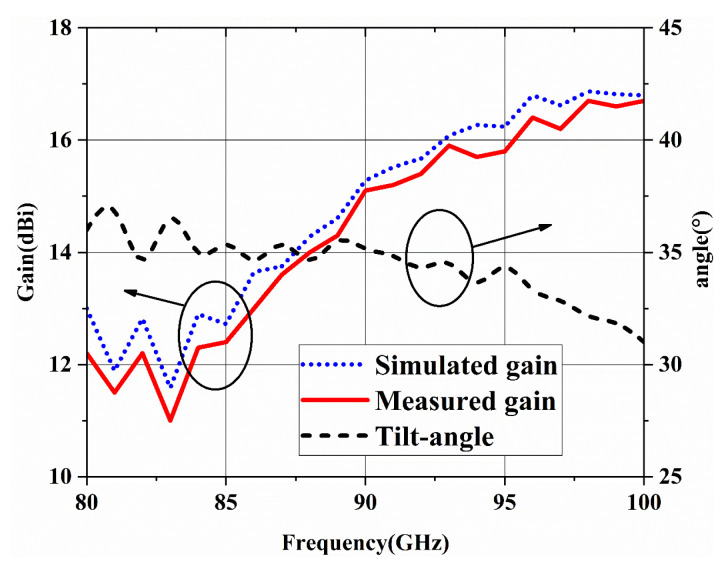
The peak gain and tilt-angle of the proposed array.

The simulated and measured radiation patterns at 80 GHz, 94 GHz and 100 GHz are shown in Figure 11. It can be seen that the simulated and measured results are in good agreement. The realized beam tilt-angles at 80 GHz, 94 GHz and 100 GHz are 36°, 33° and 31°, respectively. The gain and beam-tilted angle of proposed antenna are shown in Figure 12, the minimum antenna gain is 11.5 dBi and the maximum gain is 16.8 dBi, the fluctuation of beam-tilted is less than 4° compared with expected 35°. Possibly due to the machining tolerance, assembling or measuring errors, the measured gain is slightly lower than simulated but within the allowable margin of error. Overall speaking, although the excitation distribution is calculated at 94 GHz, the beam tilt- angle is around 35° over the whole working frequency band. This implies that the beam tilt angle has high tolerance for excitation distribution, but it also runs the risk of loss of gain.

The performance of the proposed antenna is compared with the formerly published works with the characteristic of tilted radiation beam, and the results are summarized in Table 2. Despite the antenna discussed in [5,11,12] having a bigger tilt-angle, they have a narrower bandwidth, lower radiation efficiency and unstable tilt-angle over the whole operating bandwidth. What is more, the proposed antenna integrated with feed network in this paper based on waveguide structure avoids the loss of connection and dielectric, especially in high frequency band, while maintaining wide bandwidth and comparable tilt beam angle. The realization method of the proposed antenna can be a candidate for the application at even higher frequency.

## 5. Conclusions

In this paper, a tilted-beam H-plane horn antenna array with broadband is designed. The optimized distribution of excitations for the array for different beam angles are calculated by MMPTE. As a demonstration, the antenna and the feed network are designed and fabricated for the 35° tilt angle in this paper. The feed network and antenna are manufactured by CNC-milling machines technology. According to the measured results, the feed network can generate stable amplitude with maximum fluctuation less than 1 dB, and stable phase with maximum fluctuation less than 15° from 80 GHz to 100 GHz. The measured beam pointing angle is in the range of 31° to 38° and the antenna gain changes from 11.5 dBi to 16.6 dBi in the frequency band from 80 GHz to 100 GHz.

Overall, H-plane horn antenna is verified and can be used as an element for beam control array and without grating lobe. What is more, it can easily be fabricated by the computer numerical control (CNC) milling machines. In addition, the proposed feed network can be designed to output arbitrary amplitude and phase in broadband; it lays the foundation for the later design of beam forming antenna or low sidelobe antenna based on waveguide structure. It is worth mentioning that the minimum drill radius used in the fabrication of the proposed antenna and feed network is 0.2 mm, which is much less than the current smallest drill radius of 0.05 mm. As a result, the design method and manufacture technology can be scaled up to THz band, and is likely to promote more applications of radiation beam control in the high frequency band.

## Figures and Tables

**Table 1:** 20°, 35° and 45° beam tilting angle.

Port	20°	35°	45°
2	0.19 ∠ 0°	0.19 ∠ 0°	0.18 ∠ 0°
3	0.26 ∠ 76°	0.21 ∠ −116°	0.2 ∠ −160°
4	0.23 ∠ −151°	0.29 ∠ 125°	0.28 ∠ 42°
5	0.3 ∠ 137°	0.29 ∠ 1°	0.3 ∠ −124°

**Table 2:** Comparison with published works about antenna with tilted radiation beam.

Ref.	Freq.	Tilt-Angle/Maximum Fluctuation	Gain/ Efficiency	Bandwidth	Method
[4]	60 GHz	30°/8°	12 dBi/NA	11%	Meta-lens +dipole
[5]	28 GHz	38°/8°	9 dBi/NA	10%	Metasuface +vivaldi
[11]	30 GHz	42°/5°	11.7 dBi/78%	16%	Microstrip butler +horn
[12]	77 GHz	49°/17°	12.2 dBi/NA	11%	SIW butler +slot
This work	94 GHz	35°/4°	16 dBi/98%	22%	Waveguide feed network +horn

## Data Availability

Data are available when authors are asked.

## References

[1] Dadgarpour A., Zarghooni B., Virdee B.S., Denidni T.A. (2016). Single End-Fire Antenna for Dual-Beam and Broad Beamwidth Operation at 60 GHz by Artificially Modifying the Permittivity of the Antenna Substrate. IEEE Trans. Antennas Propag..

[2] Zhang M., Matsueda S., Hirokawa J., Ando M. (2021). Realization of a Beam-Tilted Circularly Polarized Corporate-Fed Waveguide Slot Array in the 60 GHz Band. IEEE Trans. Antennas Propag..

[3] Wu G.B., Qu S.W., Yang S. (2018). Wide-Angle Beam Scanning Reflectarray with Mechanical Steering. IEEE Trans. Antennas Propag..

[4] Dadgarpour A., Zarghooni B., Virdee B.S., Denidni T.A. (2016). Improvement of Gain and Elevation Tilt Angle Using Metamaterial Loading for Millimeter-Wave Applications. IEEE Antennas Wirel. Propag. Lett..

[5] Mantash M., Kesavan A., Tahseen M.M., Denidni T.A., Kakhki M.B. (2018). Millimeter-Wave Beam-Tilting Vivaldi Antenna With Gain Enhancement Using Multilayer FSS. IEEE Antennas Wirel. Propag. Lett..

[6] Schneider D.A., Roesch M., Tessmann A., Zwick T. (2019). A Low-Loss W-Band Frequency-Scanning Antenna for Wideband Multichannel Radar Applications. IEEE Antennas Wirel. Propag. Lett..

[7] Bhattacharjee A., Dwari S. (2021). A Monopole Antenna With Reconfigurable Circular Polarization and Pattern Tilting Ability in Two Switchable Wide Frequency Bands. IEEE Antennas Wirel. Propag. Lett..

[8] Ouyang W., Gong X. (2020). A 20-Element Cavity-Backed Slot Electronically Steerable Parasitic Array Radiator (ESPAR) With 2-D Beamsteering and Minimized Beam Squint. IEEE Antennas Wirel. Propag. Lett..

[9] Jia Q., Xu H., Xiong M.F., Zhang B., Duan J. (2019). Omnidirectional Solid Angle Beam-switching Flexible Array Antenna in Millimeter Wave for 5G Micro Base Station Applications. IEEE Access.

[10] Kong W., Hu Y., Li J., Zhang L., Hong W. (2022). 2-D Orthogonal Multibeam Antenna Arrays for 5G Millimeter-Wave Applications. IEEE Trans. Microw. Theory Tech..

[11] Ashraf N., Sebak A.R., Kishk A.A. (2020). PMC Packaged Single-Substrate 4 × 4 Butler Matrix and Double-Ridge Gap Waveguide Horn Antenna Array for Multibeam Applications. IEEE Trans. Microw. Theory Tech..

[12] Djerafi T., Wu K. (2012). A Low-Cost Wideband 77-GHz Planar Butler Matrix in SIW Technology. IEEE Trans. Antennas Propag..

[13] Wen G. (2021). The Method of Maximum Power Transmission Efficiency for the Design of Antenna Arrays. IEEE Open J. Antennas Propag..

[14] Xiao C., Wen G. (2019). An Optimization Method for the Synthesis of Flat-Top Radiation Patterns in the Near- and Far-Field Regions. IEEE Trans. Antennas Propag..

[15] Xiao C., Wen G., Sun H. (2017). A New Printed Dipole Array With High Gain and End-Fire Radiation. IEEE Antennas Wirel. Propag. Lett..

[16] Wyndrum (1964). Microwave filters, impedance-matching networks, and coupling structures. Proc. IEEE.

[17] Reed J. (1958). The Multiple Branch Waveguide Coupler. Microw. Theory Tech. Ire Trans..

[18] Ding J., Yun Z., Ge J.X., Shi S. (2019). A 90° Waveguide Hybrid with Low Amplitude Imbalance in Full W -Band. J. Infrared Millim. Terahertz Waves.

[19] Liu R., Hu W., Hao W., Wang X., Wang Y. Design of a 220GHz power divider with T-shape configuration. Proceedings of the 2016 IEEE 9th UK-Europe-China Workshop on Millimetre Waves and Terahertz Technologies (UCMMT).

[20] Su T., Yu C., Zhao M. W-band four-way E-plane waveguide power divider. Proceedings of the IEEE Mtt-s International Microwave Workshop Series on Advanced Materials & Processes for Rf & Thz Applications.

[21] Yang Y.M., Yuan C.W., Cheng G.X., Qian B.L. (2015). Ku-Band Rectangular Waveguide Wide Side Dimension Adjustable Phase Shifter. IEEE Trans. Plasma Sci..

